# 1,2-Difluoroethylene (HFO-1132): synthesis and chemistry

**DOI:** 10.3762/bjoc.20.171

**Published:** 2024-08-12

**Authors:** Liubov V Sokolenko, Taras M Sokolenko, Yurii L Yagupolskii

**Affiliations:** 1 Institute of Organic Chemistry, National Academy of Sciences of Ukraine, Academician Kukhar str., 5, Kyiv-94, 02660, Ukraine

**Keywords:** 1,2-difluoroethylene, fluorinated monomers, HFO-1132, hydrofluoroolefins, radical reactions, refrigerants

## Abstract

This article provides a comprehensive overview of the synthesis and chemistry of 1,2-difluoroethylene (HFO-1132). The major routes for the preparation of the *E*- and *Z*-isomer of HFO-1132 are reviewed, along with the chemistry in radical, nucleophilic, and electrophilic reactions.

## Introduction

In the 1930s, halogenated chlorofluorocarbons (CFCs) and hydrochlorofluorocarbons (HCFCs) were synthesized and have been shown to have low toxicity, which has opened the door for the application as safe refrigerants [1–2]. The development of the commercial synthesis of CFCs and HCFCs, along with new refrigeration systems in the 1930s–1960s, has led to the wide application of these materials in household and commercial refrigeration systems [1–2]. In addition, CFCs have found applications as propellants, foam-blowing agents, cleaning solvents, etc. Although these groups of fluorinated materials are non-flammable and have low toxicity, CFCs were found to be destructive to the ozone layer of the stratosphere [1–2] due to high ozone-depleting potential (ODP). This has led to the phasing out of CFCs and the replacement with hydrofluorocarbons (HFCs), which show no significant impact on stratospheric ozone [1–3]. However, as it was demonstrated in the 1980s, HFCs have significant global warming potential (GWP) [1–3]. In the 2000s, a new generation of refrigerants, namely hydrofluoroolefins (HFOs), which have a short atmospheric lifetime and low GWP [4–5], has been introduced into commercial use [1–3]. Recently, these compounds and blends thereof have replaced HFCs in refrigerants and air-conditioning systems [1–3].

The commercial production of HFOs has made these compounds available for chemists to be used as important reagents in laboratories. For example, the interest in CF_3_CH=CHCF_3_ (HFO-1336mzz) [6–12], CF_3_CH=CHCl (HCFO-1233zd) [13–18], CF_3_CH=CHF (HFO-1234ze) [12,19–23], and CF_3_CF=CH_2_ (HFO-1234yf) [12,20–21,23–39] as fluorinated building blocks has significantly increased in the last years.

Recently, the *E*-isomer of 1,2-difluoroethylene ((*E*)-HFO-1132) has attracted attention as a new refrigerant due to the low boiling point, moderate flammability, and low toxicity [40–42]. Also, in patent literature 1,2-difluoroethylene has been claimed to be a potential monomer for the preparation of new fluoropolymers [43–44].

Despite the fact that HFO-1132 has been known for a long time, there are no publications that summarize the chemistry of the compound. With this in mind, the main methods for the preparation of (*E*/*Z*)-1,2-difluoroethylene are discussed in this Review article. Special attention is given to the role of 1,2-difluoroethylene in multiple reaction types.

## Review

### Preparation of HFO-1132

**Scheme 1:**
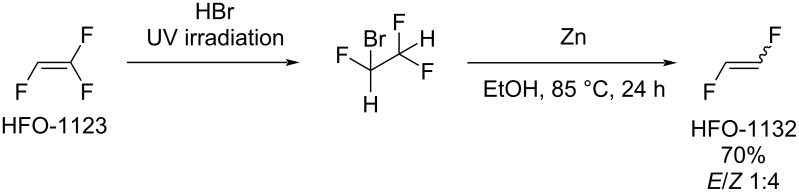
1,2-Difluoroethylene synthesis from HFO-1123.

In scientific literature, the number of publications on the synthesis of 1,2-difluoroethylene is limited. HFO-1132 was first obtained in 1955 as a byproduct in the reaction of diborane with tetrafluoroethylene [45]. To the best of our knowledge, the first preparative route to 1,2-difluoroethylene was described in 1957 by Haszeldine and Steele [46], using trifluoroethylene as starting material (Scheme 1) [46–47].

**Scheme 2:**
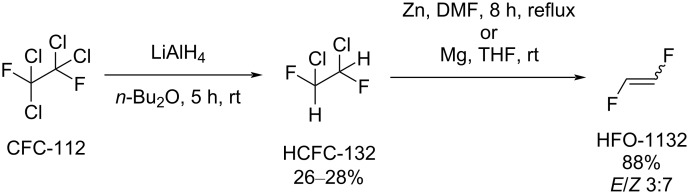
1,2-Difluoroethylene synthesis from CFC-112 and HCFC-132.

Another approach to 1,2-difluoroethylene was based on 1,2-dichloro-1,2-difluoroethane (HCFC-132) [48–52], prepared from 1,1,2,2-tetrachloro-1,2-difluoroethane (CFC-112) by reduction using lithium aluminum hydride [48–51] or photoreduction (Scheme 2) [51]. The resulting HCFC-132 reacted with zinc [47,49,52] or magnesium [50–51] to form the desired 1,2-difluoroethylene as a mixture of *E*- and *Z*-isomers, which were separated by fractional distillation (boiling point: −53.1 °C for (*E*)-HFO-1132 and −26.0 °C for (*Z*)-HFO-1132 [47]).

**Scheme 3:**
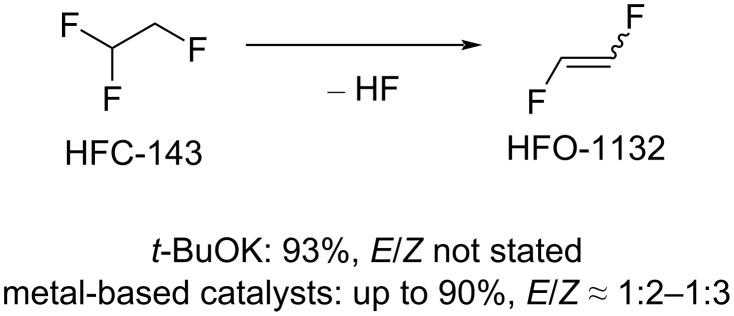
1,2-Difluoroethylene synthesis from HFC-143.

In patent literature, a variety of synthetic routes to HFO-1132 starting from other available industrial halocarbons can be found. It should be pointed out that the majority of patents in this field belongs to Daikin Industries [53–60]. Scheme 3 shows the synthesis of HFO-1132 based on dehydrofluorination of 1,1,2,-trifluoroethane (HFC-143) in the presence of base (e.g., *t*-BuOK) [53] or metal (Cr, Al, Fe, Ni, Mg)-based catalyst [54–56].

**Scheme 4:**
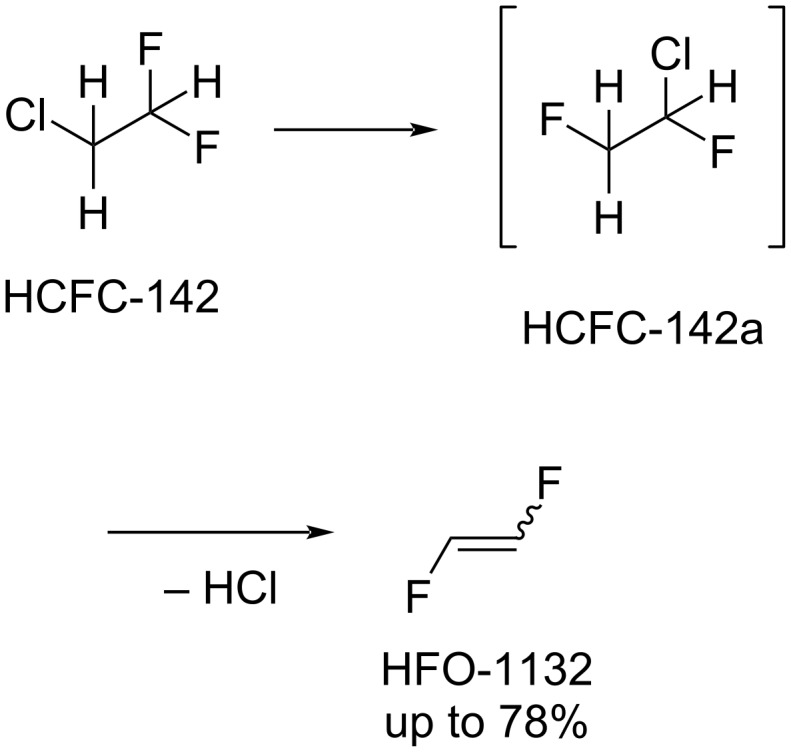
1,2-Difluoroethylene synthesis from HCFC-142 via HCFC-142a.

2-Chloro-1,2-difluoroethane (HCFC-142) and 1-chloro-1,2-difluoroethane (HCFC-142a) can also be used as 1,2-difluoroethene precursors (Scheme 4) [57,61]. The dehydrochlorination reaction proceeded in the presence of metal-based catalysts (Fe, Mg, Ca, Ni, Zn, Pd, Li, Na [57] or Pd, Ru [61]).

**Scheme 5:**
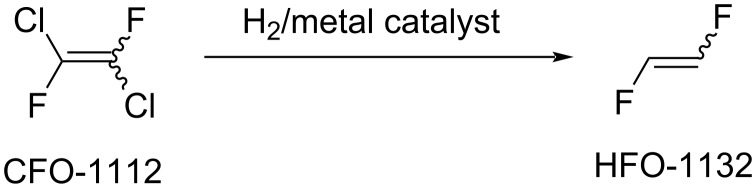
1,2-Difluoroethylene synthesis from CFO-1112.

**Scheme 6:**
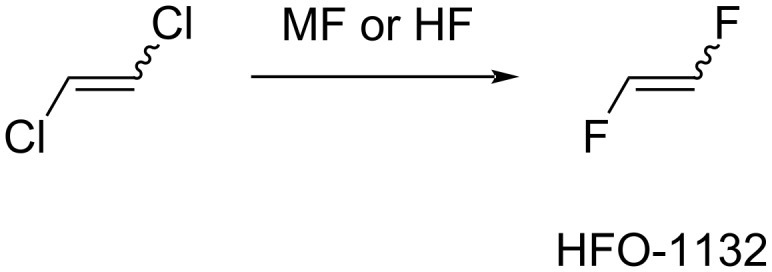
1,2-Difluoroethylene synthesis from 1,2-dichloroethylene.

Unsaturated compounds were also applied as starting materials for 1,2-difluoroethylene preparation. Thus, chlorine atoms in 1,2-dichloro-1,2-difluoroethylene (CFO-1112) could be removed through the action of hydrogen and metal catalyst (Pd, Pd, Pt, Rh, Ru, Ir, Ni/Cu, Ag, Au, Zn, Cr, Co, Scheme 5) [62–63]. Further, 1,2-Dichloroethylene was reacted with hydrogen fluoride in the presence of metal fluorides or transition metals (Cr, Al, Co, Mn, Ni, Fe) to form 1,2-difluoroethylene (Scheme 6) [56,58].

**Scheme 7:**
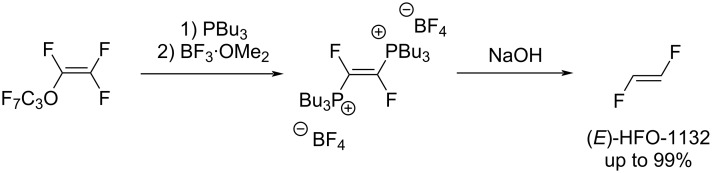
1,2-Difluoroethylene synthesis from perfluoropropyl vinyl ether.

In patents [59–60], an exotic synthesis of 1,2-difluoroethylene based on perfluoropropyl vinyl ether as starting material can be found (Scheme 7).

Consequently, two methods to prepare 1,2-difluoroethylene in the laboratory have been described to date. Even though at least five approaches to HFO-1132 can be found in patent literature, it is not clear which of these can be used for the commercial production of HFO-1132.

### Physical properties of HFO-1132

The physical properties of the *E*- and *Z*-isomers of HFO-1132 are summarized in Table 1 [47,64–66].

**Table 1:** Physical properties of HFO-1132.

parameter	(*E*)-HFO-1132	(*Z*)-HFO-1132

boiling point, °C	−53.1 [47]	−26.0 [47]
density (at −153 °C), g/cm^3^	1.592 [64]	1.556 [64]
dipole moment	0 [65]	2.81 (calculated) [65] 2.42 (experimental) [65]
ODP	0 [40]	—
GWP_100_^a^	1.9 [66]	1.5 [66]

^a^Average GWP over 100 years.

IR-spectral data of (*E*)- and (*Z*)-HFO-1132 can be found in references [67] and [50], respectively, while reference [68] provides UV-spectral data of both isomers. ^1^H, ^19^F, and ^13^C NMR data [69–70] are given in Table 2.

**Table 2:** NMR-spectral data of HFO-1132.

compound	^1^H NMR (300 MHz, C_6_D_6_, δ), ppm [69]	^19^F NMR (282 MHz, C_6_D_6_, δ), ppm [69]	^13^C NMR^1^ (δ), ppm [70]

(*E*)-HFO-1132	6.68 (m)	−187.73 (dd, ^2^*J*_FH_ = 49 Hz, ^3^*J*_FH_ = 30 Hz)	146.0
(*Z*)-HFO-1132	5.54 (m)	−163.09 (m)	138.5

^1^CH_4_ as standard.

### Chemistry of HFO-1132

#### Isomerization

Iodine-catalyzed *cis*–*trans* isomerization of 1,2-difluoroethylene and corresponding equilibrium measurements were described in the 1960s [47]. Along with this, photoisomerization is described in patent literature [71–74]. It was shown that the experimentally observed enthalpy of isomerization (0.928 kcal/mol [47]) is in agreement with the calculated difference in the total energy of the two isomers (0.959 kcal/mol [65,75]). *Cis*-1,2-difluoroethylene was shown to have a lower energy compared to *trans*-1,2-difluoroethylene, which is in accordance with previously described 1,2-dihalogenated ethylene species [47].

The authors of reference [47] explained the higher stability of (*Z*)-HFO-1132 as follows: Within the family of 1,2-dihaloethylenes, when going from diiodo- and dibromo- to dichloro- and difluoroethylene, the radius of the halogen atom decreases while the electronegativity increases. As a result, the influence of halogen atom electronegativity on the double bond is more significant in 1,2-difluoroethylene, and the relative energy of the *cis*-isomer decreases, i.e., the *cis*-isomer of 1,2-difluoroethylene is thermodynamically favored [47].

#### Deuteration

**Scheme 8:**
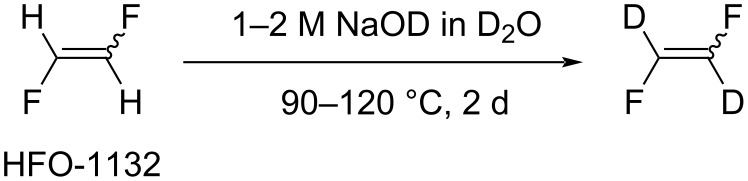
Deuteration reaction of 1,2-difluoroethylene.

The stereospecific reaction of (*E*/*Z*)-1,2-difluoroethylene with a 1–2 M solution of NaOD in D_2_O (90–120 °C, 2 d) led to the formation of CDF=CDF with high isotopic purity (Scheme 8) [76–77].

Additionally, when the reaction was performed using DMSO-*d*_6_ (or CD_3_CN) and CH_3_ONa, H/D exchange occurred already at ambient temperature (25 °C, 20 h) [78]. The formation of CDF=CDF was confirmed by NMR spectroscopy, namely by the change of signal multiplicity in the ^19^F NMR spectra of *E*- and *Z*-isomers of 1,2-difluoroethylene and the disappearance of vinyl protons resonances in the ^1^H NMR spectra [78].

#### Addition to the C=C bond

**Scheme 9:**
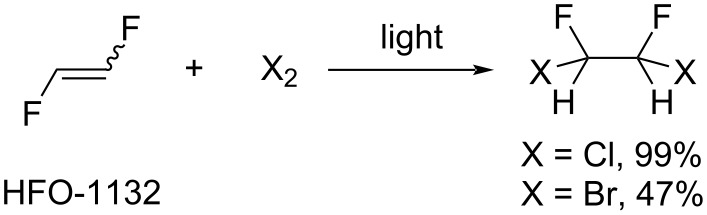
Halogen addition to 1,2-difluoroethylene.

**Halogen addition:** 1,2-Difluoroethylene was reported to react with chlorine [46,79] and bromine [51] under irradiation, yielding 1,2-difluoro-1,2-dihaloethanes in moderate to high yield (Scheme 9).

**Scheme 10:**
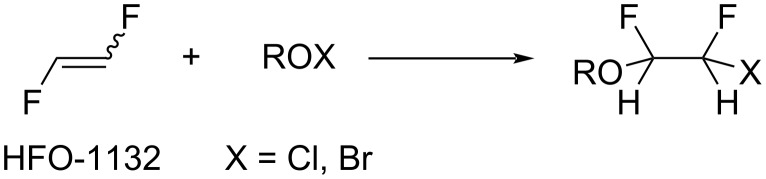
Hypohalite addition to 1,2-difluoroethylene.

**Hypohalite addition:** It was shown by the DesMarteau group that different hypohalites (perfluoroalkyl-, perfluoroacyl-, perfluoroalkylsulfonyl-, and peroxyhypochlorite) easily reacted with 1,2-difluoroethylene to form addition products in high to quantitative yield (Scheme 10) [80–88]. In Table 3, reaction data are summarized to show the scope and limitations of this process.

**Table 3:** Hypohalite addition to 1,2-difluoroethylene.

entry	hypohalite	configuration	product	yield, %	Reference

1	CF_3_CF_2_C(CF_3_)_2_OBr	*Z* (*cis*)	*threo*-CF_3_CF_2_C(CF_3_)_2_OCFH–CFHBr	74	[81]
2	FSO_2_CF_2_CF_2_OCl	*Z* (*cis*)	*erythro*-FSO_2_CF_2_CF_2_OCFH–CFHCl	100	[80]
3	CF_3_OF	*Z* (*cis*)	CF_3_OCFH–CF_2_H	100	[82]
4	CF_3_OCl	*Z* (*cis*)	*erythro*-CF_3_OCFH–CFHCl	86	[82]
		*E* (*trans*)	*threo*-CF_3_OCFH–CFHCl	88	
		*E*/*Z* 8:5	*erythro*/*threo*-CF_3_OCFH–CFHCl 8:5	not stated	
5	CF_3_C(O)OCl	*Z* (*cis*)	*erythro*-CF_3_C(O)OCFH–CFHCl	64	[83]
		*E* (*trans*)	*threo*-CF_3_C(O)OCFH–CFHCl	65	
6	CF_3_SO_2_OCl	*Z* (*cis*)	*erythro*-CF_3_SO_2_OCFH–CFHCl	88	[84–85]
		*Z*/*E* 3:2	*erythro*/*threo*-CF_3_SO_2_OCFH–CFHCl ≈ 3:2	90	
7	CF_3_SO_2_OBr	*Z* (*cis*)	*erythro*-CF_3_SO_2_OCFH–CFHBr	87	[84]
		*Z*/*E* 3:2	*erythro*/*threo*-CF_3_SO_2_OCFH–CFHBr ≈ 3:2	95	
8	C_4_F_9_SO_2_OCl	*Z* (*cis*)	*erythro*-C_4_F_9_SO_2_OCFH–CFHCl	80	[86]
9	C_4_F_9_SO_2_OBr	*Z* (*cis*)	*erythro*-C_4_F_9_SO_2_OCFH–CFHBr	80	[86]
10	CF_3_OOCl	*Z* (*cis*)	*erythro*-CF_3_OOCFH–CFHCl	40^a^	[87]
11	SF_5_OOCl	*Z* (*cis*)	*erythro*-SF_5_OOCFH–CFHCl	70	[88]

^a^Chloroperoxytrifluoromethane is an unstable compound that decomposed to CF_3_OCl. Therefore, CF_3_OCFH–CFHCl byproduct was also isolated in 11% yield in this reaction.

An interesting feature of this reaction is the high stereospecificity. In almost all cases, the addition proceeded *syn*-specific, yielding the *erythro*-isomer from *cis*- and the *threo*-isomer from *trans*-1,2-difluoroethylene, respectively, with one exception: *threo*-isomer formation from *cis*-1,2-difluoroethylene (entry 1, Table 3). Supposedly this was due to dominant steric factors, such that the reaction occurred as *anti*-addition.

**Scheme 11:**
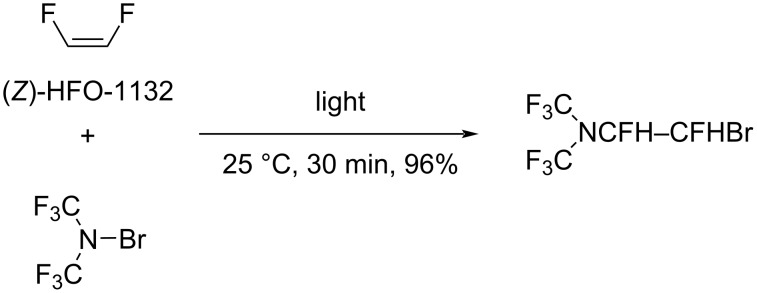
*N*-Bromobis(trifluoromethyl)amine addition to 1,2-difluoroethylene.

**Addition of *N*-halo compounds:** It was shown by Haszeldine and Tipping that *N*-bromobis(trifluoromethyl)amine easily reacted with (*Z*)-1,2-difluoroethylene to form the addition product in high yield (Scheme 11) [89]. However, the stereochemistry of this reaction has not been reported.

**Scheme 12:**
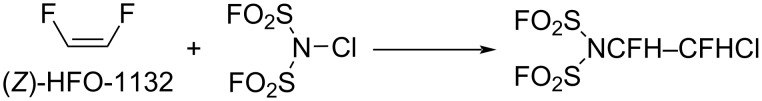
*N*-Chloroimidobis(sulfonyl fluoride) addition to 1,2-difluoroethylene.

A similar reaction of (*Z*)-1,2-difluoroethylene with *N*-chloroimidobis(sulfonyl fluoride) (Scheme 12) [90] was shown to be stereounspecific, although the addition product was reported to form in high yield.

In the same publication [90], it was mentioned that (FSO_2_)_2_NH did not form an addition product in the reaction with (*Z*)-1,2-difluoroethylene, although the reaction of (FSO_2_)_2_NH with other olefins, including fluorinated ones, occurred similar to HF addition [90].

**Scheme 13:**
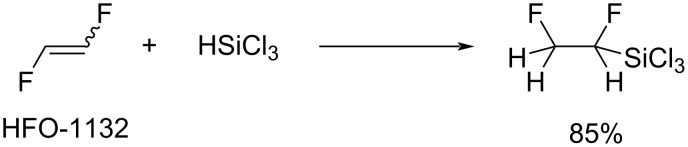
Trichlorosilane addition to 1,2-difluoroethylene.

**Miscellaneous additions:** In reference [91], the addition of trichlorosilane to 1,2-difluoroethylene (Scheme 13) was reported by the Haszeldine group. The reaction under UV irradiation produced the corresponding trichlorosilane in 85% yield, and the silane that was obtained was pyrolyzed to form vinyl fluoride.

**Scheme 14:**
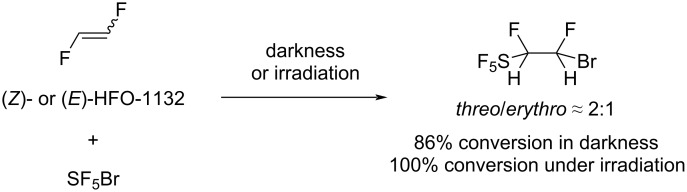
SF_5_Br addition to 1,2-difluoroethylene.

It was shown that SF_5_Br easily reacted with the *E*- and *Z*-isomer, respectively, of 1,2-difluoroethylene in the presence or absence of light, yielding a mixture of *erythro*- and *threo*-isomeric addition products in both cases (Scheme 14) [92]. However, under light irradiation, conversion and product yield were higher, although the ratio of diastereomers produced in both cases was almost the same for the *E*- and *Z*-isomer, respectively, and did not depend on irradiation.

**Scheme 15:**
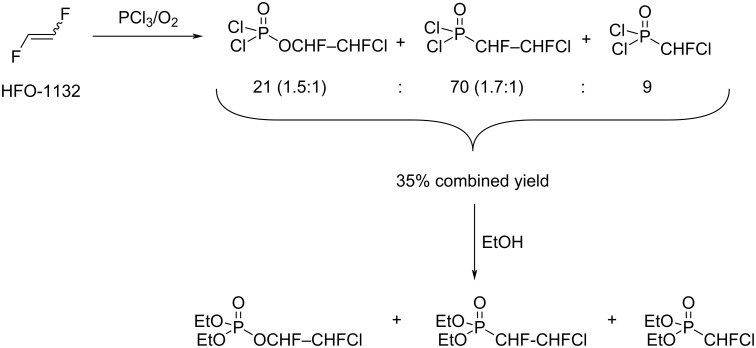
PCl_3_/O_2_ addition to 1,2-difluoroethylene.

The reaction of 1,2-difluoroethylene with PCl_3_ and O_2_ was described by Boyce and co-workers [93]. Therein, a mixture of products, with diethyl 2-chloro-1,2-difluoroethylphosphonate as main compound, was formed (Scheme 15). This mixture was reacted with absolute ethanol, and the esters formed were separated by distillation and characterized. The authors did not point out which 1,2-difluoroethylene isomer (*E* and/or *Z*) was used. It was mentioned that the addition products were obtained as a mixture of diastereomers (Scheme 15).

**Scheme 16:**
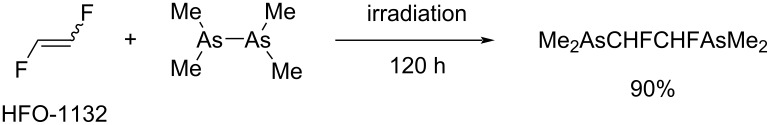
Reaction of tetramethyldiarsine with 1,2-difluoroethylene.

Tetramethyldiarsine was shown to react with (*Z*/*E*)-1,2-difluoroethylene under UV irradiation, yielding the product as a mixture of the racemate and the *meso* form in high combined yield (90%, Scheme 16) [94]. The product was used as a ligand for the preparation of transitional metal carbonyl complexes.

**Scheme 17:**
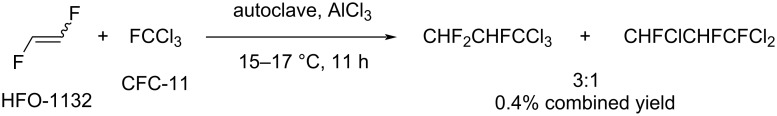
Reaction of trichlorofluoromethane with 1,2-difluoroethylene.

The addition reaction of trichlorofluoromethane (CFC-11) to 1,2-difluoroethylene in the presence of aluminum chloride under pressure was described [51]. In this electrophilic reaction, two products were formed in 3:1 ratio (Scheme 17) in a very low yield of 0.4%.

**Scheme 18:**
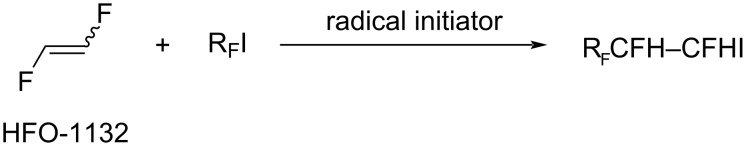
Addition of perfluoroalkyl iodides to 1,2-difluoroethylene.

In patent literature [95], radical reaction of 1,2-difluoroethylene with long-chain perfluoroalkyl iodides (C*_n_*F_2_*_n_*_ + 1_I, *n* = 2–8) was described (Scheme 18). Products formed were further converted into polyfluorinated olefins R_F_CF=CFH by HI elimination.

#### Cyclization reactions

**Scheme 19:**
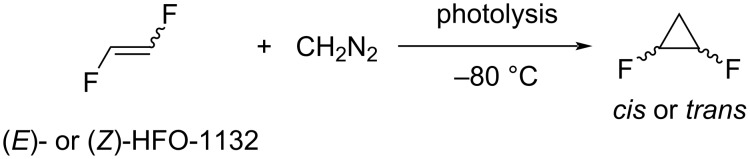
Cyclopropanation of 1,2-difluoroethylene.

**Carbocyclizations:** A series of articles devoted to structural investigations of 1,2-difluorocyclopropanes was published [96–98]. For this purpose, *cis*- and *trans*-1,2-difluorocyclopropanes were synthesized by liquid-phase photolysis at −80 °C from 1,2-difluoroethylene and diazomethane (Scheme 19). Unfortunately, the product yield was not reported.

**Scheme 20:**
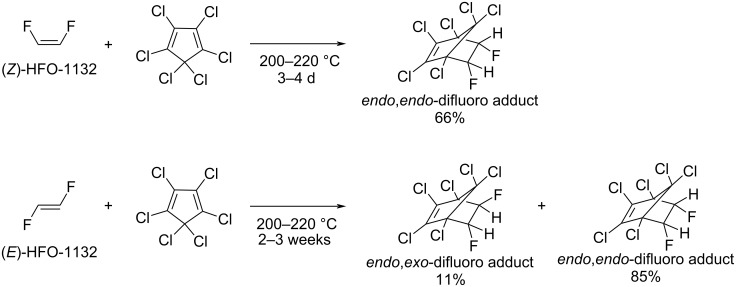
Diels–Alder reaction of 1,2-difluoroethylene and hexachlorocyclopentadiene.

Diels–Alder reaction of (*E*)- and (*Z*)-1,2-difluoroethylenes with hexachlorocyclopentadiene was studied by Ihrid and Smith [99]. It was shown that (*Z*)-1,2-difluoroethylene reacted with hexachlorocyclopentadiene at 200–220 °C within 3–4 days, forming 5,6-*endo*,*endo*-difluoro-1,2,3,4,7,7-hexachlorobicyclo[2.2.1]-2-heptene (Scheme 20) in 66% yield. At the same time, (*E*)-1,2-difluoroethylene, which formed the *endo*,*exo*-adduct, reacted much slower, and completion of the reaction required 2–3 weeks at the same temperature. In this case, sufficient *E*–*Z* isomerization of the starting olefin occurred during the reaction, and the major product was 5,6-*endo*,*endo*-difluoro-1,2,3,4,7,7-hexachlorobicyclo[2.2.1]-2-heptene, which was formed from (*Z*)-1,2-difluoroethylene (Scheme 20). Both products were separated by column chromatography and characterized.

**Scheme 21:**
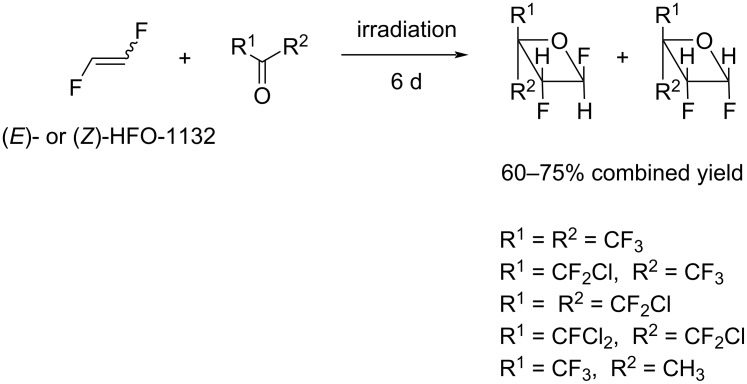
Cycloaddition reaction of 1,2-difluoroethylene and fluorinated ketones.

**Heterocyclizations–photochemical [2 + 2]- and [2 + 4]-cycloaddition reactions:** The formation of oxetanes as a result of photochemical cycloaddition of fluoroketones or fluoroaldehydes and 1,2-difluoroethylene was previously described by Haszeldine et al. [49,100]. The reaction of individual *E*- or *Z*-isomer of 1,2-difluoroethylene and fluorinated ketones (Scheme 21) led to a mixture of stereoisomers in both cases, although for the *E*-isomer, about 70% of the product retained the starting configuration. Overall, (*E*)-1,2-difluoroethylene had higher reactivity than (*Z*)-1,2-difluoroethylene in this reaction.

**Scheme 22:**
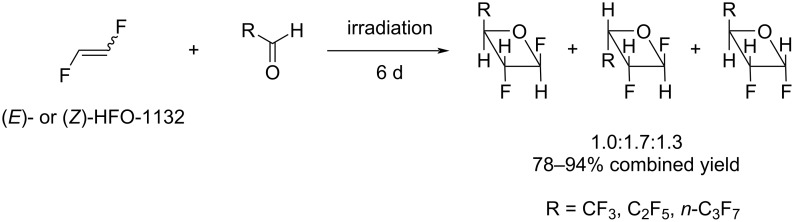
Cycloaddition reaction of 1,2-difluoroethylene and perfluorinated aldehydes.

The reaction of either (*E*)- or (*Z*)-1,2-difluoroethylene with perfluoroaldehydes resulted in the formation of three isomeric oxetanes in a 1.0:1.7:1.3 ratio in a high yield of 78–94% (Scheme 22) [49].

All data reported for the [2 + 2]-cycloaddition reaction of fluorinated ketones and aldehydes [49,100] were indicative of the fact that under photochemical conditions, this reaction is likely to be a stepwise process involving the formation of a biradical intermediate.

**Scheme 23:**
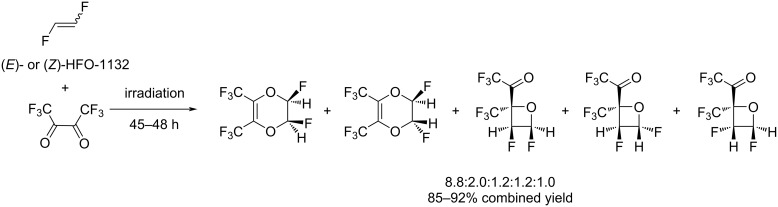
Photochemical cycloaddition of 1,2-difluoroethylene and hexafluorodiacetyl.

Either (*Z*)- or (*E*)-1,2-difluoroethylene easily reacted with hexafluorodiacetyl under UV irradiation, yielding a mixture of five products, regardless of the configuration of the starting 1,2-difluoroethylene, in a ratio of 8.8:2.0:1.2:1.2:1.0 in 85% and 92% yield for the *Z*- and *E*-olefin, respectively (Scheme 23) [48]. Interestingly, the formation of [4 + 2]-adducts in this case was predominant over [2 + 2]-cycloadducts.

#### Reactions involving C–F bonds

**Scheme 24:**
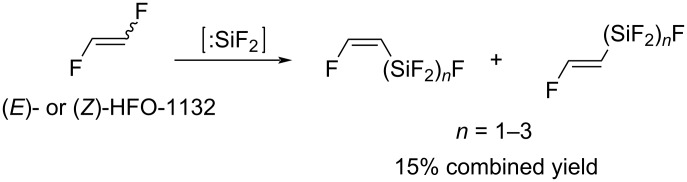
Reaction of 1,2-difluoroethylene with difluorosilylene.

It was shown by Liu and co-workers that SiF_2_ was able to insert into the C–F bond of 1,2-difluoroethylene, as well as into the emerging Si–F bond, leading to a mixture of fluoropolysilanes with a low combined yield (Scheme 24) [101–102].

**Scheme 25:**
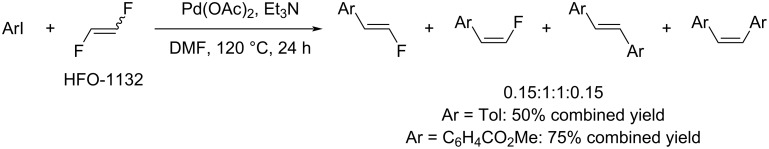
Reaction of 1,2-difluoroethylene with aryl iodides.

Our group attempted to use (*E*/*Z*)-1,2-difluoroethylene in a Heck reaction [78]. The experiments were performed using 4-iodotoluene or methyl 4-iodobenzoate in DMF, Pd(OAc)_2_ as a catalyst, and Et_3_N as a base (Scheme 25). The reactions were carried out in a stainless steel autoclave at 120 °C for 24 h. Careful investigation of the product structures by ^1^H and ^19^F NMR as well as GC–MS revealed exclusive substitution of fluorine rather than hydrogen, leading to a mixture of products in the ratio 0.15:1:1:0.15 (Scheme 25), with a combined yield of 50% for 4-iodotoluene and 75% for methyl 4-iodobenzoate. Under similar conditions, 4-nitroiodobenzene produced exclusively the corresponding homocoupling product 4,4’-nitrobiphenyl.

#### Additional author remarks

Other attempts to utilize 1,2-difluoroethylene in reactions with N-, O-, and C- nucleophiles carried out in our group were unsuccessful [78], while S-nucleophiles, namely thiophenolates, led to products upon fluorine atom substitution, which were isolated in low yield. Corresponding disulfides were isolated as major products, even when the reaction was carried out under inert atmosphere, suggesting a radical process.

In summary, we compiled the methods for the preparation of HFO-1132 as well as reactions demonstrating the chemical behavior of this compound. From the reactions not included in this Review article, mechanistic studies on 1,2-difluoroethylene ozonolysis [77,103–108] and studies on the stability of transitional metal complexes with 1,2-difluoroethylene as a ligand should be mentioned [109–111].

## Conclusion

In conclusion, our literature analysis demonstrated that radical processes are most typical for 1,2-difluoroethylene, while examples of electrophilic reactions are scarce, and nucleophilic reactions were not described at all. Nevertheless, the radical reactions are the most powerful instrument for the preparation of new molecules with a CHF–CHF fragment. For instance, the radical addition of hypohalites is a suitable high-yielding approach toward polyfluorinated aliphatic ethers and esters. Photochemical [2 + 2]-cycloaddition with fluorinated aldehydes and ketones gives access to a variety of fluorinated oxygen-containing heterocycles. We hope that this article will help chemists to utilize HFO-1132 and that this olefin will find applications as a useful synthon in organic chemistry.

## Data Availability

Data sharing is not applicable as no new data was generated or analyzed in this study.
